# Phenotypic and molecular characterization of β-lactamase-producing *Klebsiella* species among children discharged from hospital in Western Kenya

**DOI:** 10.1186/s12866-024-03284-7

**Published:** 2024-04-23

**Authors:** Doreen Rwigi, Andrew K. Nyerere, Mame M. Diakhate, Kevin Kariuki, Kirkby D. Tickell, Timothy Mutuma, Stephanie N. Tornberg, Olusegun O. Soge, Judd L. Walson, Benson Singa, Samuel Kariuki, Patricia B. Pavlinac, Polycarp Mogeni

**Affiliations:** 1 Kenya Medical Research Institute (KEMRI), Nairobi, Kenya; 2 Center for Microbiology Research (CMR), Kenya Medical Research Institute (KEMRI), Nairobi, Kenya; 3 Jomo Kenyatta University of Agriculture and Technology, Nairobi, Kenya; 4 Department of Global Health, University of Washington, Seattle, Washington USA; 5 Department of Medicine, Division of Allergy and Infectious Diseases, University of Washington, Seattle, Washington USA; 6 Department of Laboratory Medicine and Pathology, University of Washington, Seattle, Washington, USA; 7 The Childhood Acute Illness & Nutrition (CHAIN) Network, Nairobi, Kenya; 8 Department of Epidemiology, University of Washington, Seattle, Washington USA; 9 Department of Pediatrics, University of Washington, Seattle, Washington USA; 10 Department of International Health, Johns Hopkins University, Baltimore, MD USA

**Keywords:** Antimicrobial resistance, Beta-lactams, *Klebsiella* spp, Extended Spectrum Beta lactamases, Cephalosporins

## Abstract

**Background:**

The emergence and spread of β-lactamase-producing *Klebsiella* spp. has been associated with a substantial healthcare burden resulting in therapeutic failures. We sought to describe the proportion of phenotypic resistance to commonly used antibiotics, characterize β-lactamase genes among isolates with antimicrobial resistance (AMR), and assess the correlates of phenotypic AMR in *Klebsiella* spp. isolated from stool or rectal swab samples collected from children being discharged from hospital.

**Methods:**

We conducted a cross-sectional study involving 245 children aged 1–59 months who were being discharged from hospitals in western Kenya between June 2016 and November 2019. Whole stool or rectal swab samples were collected and *Klebsiella* spp. isolated by standard microbiological culture. β-lactamase genes were detected by PCR whilst phenotypic antimicrobial susceptibility was determined using the disc diffusion technique following standard microbiology protocols. Descriptive analyses were used to characterize phenotypic AMR and carriage of β-lactamase-producing genes. The modified Poisson regression models were used to assess correlates of phenotypic beta-lactam resistance.

**Results:**

The prevalence of β-lactamase carriage among *Klebsiella* spp. isolates at hospital discharge was 62.9% (154/245). Antibiotic use during hospitalization (adjusted prevalence ratio [aPR] = 4.51; 95%CI: 1.79–11.4, *p* < 0.001), longer duration of hospitalization (aPR = 1.42; 95%CI: 1.14–1.77, *p* < 0.002), and access to treated water (aPR = 1.38; 95%CI: 1.12–1.71, *p* < 0.003), were significant predictors of phenotypically determined β-lactamase. All the 154 β-lactamase-producing *Klebsiella* spp. isolates had at least one genetic marker of β-lactam/third-generation cephalosporin resistance. The most prevalent genes were *bla*_CTX-M_ 142/154 (92.2%,) and *bla*_SHV_ 142/154 (92.2%,) followed by *bla*_TEM_ 88/154 (57.1%,) and *bla*_OXA_ 48/154 (31.2%,) respectively.

**Conclusion:**

Carriage of β-lactamase producing *Klebsiella* spp. in stool is common among children discharged from hospital in western Kenya and is associated with longer duration of hospitalization, antibiotic use, and access to treated water. The findings emphasize the need for continued monitoring of antimicrobial susceptibility patterns to inform the development and implementation of appropriate treatment guidelines. In addition, we recommend measures beyond antimicrobial stewardship and infection control within hospitals, improved sanitation, and access to safe drinking water to mitigate the spread of β-lactamase-producing *Klebsiella* pathogens in these and similar settings.

**Supplementary Information:**

The online version contains supplementary material available at 10.1186/s12866-024-03284-7.

## Background

Antimicrobial resistance (AMR) is a leading cause of death worldwide, with the highest burden reported in sub-Saharan Africa (SSA) where approximately 1.2 million deaths in 2019 were attributed to bacterial AMR [1, 2]. Nearly all AMR deaths related to under 5-year-old occur in low- or middle-income countries (LMICs) [3] where AMR is associated with a substantial healthcare burden resulting from empirical antimicrobial treatment failure [4]. This translates to a considerable disease burden resulting from limited treatment options, more severe disease leading to longer duration of hospitalization, poorer clinical outcomes, and increased healthcare associated costs [3].

*Klebsiella* spp. are gram-negative commensal bacteria with pathogenic potential commonly found in the gut. *Klebsiella* spp. bacteremia, for example, has a case fatality rate of at least 30% [5]. Resistance to a commonly used class of antibiotics, beta-lactams, as measured by extended spectrum beta-lactamases (ESBLs), is associated with a 50% higher case fatality rates [5]. ESBLs are of public health concern because they not only suggests resistance to an entire class of antibiotics but can facilitate selection for resistance determinants in other antimicrobial classes, including aminoglycosides and fluoroquinolones [6]. According to Kenyan guidelines [7], initial treatments for suspected severe bacterial infections involve the use of two beta-lactam antibiotics, penicillin, or ampicillin, along with the aminoglycoside antibiotic gentamicin. Subsequently, intravenous ceftriaxone, a cephalosporin antibiotic, is recommended [8]. However, cephalosporin-resistant Klebsiella infections has posed challenges with these antibiotic regimens [8]. ESBL-producing *Klebsiella* spp. is a growing problem in SSA, where antibiotic options are already limited.

Nosocomial spread of *Klebsiella* spp. is prevalent, especially in crowded hospital environments where children are frequently exposed to antibiotics. This hospital settings serves as a particularly significant breeding ground for antibiotic resistant *Klebsiella* spp. Children returning home from hospital who develop infection with AMR *Klebsiella* may have limited treatment options and may spread these AMR bacteria within households and the community.

Although AMR bacterial infections pose a disproportionate public health challenge in SSA, epidemiological data are scarce. We conducted an AMR study that was nested within a clinical trial investigating the impact of azithromycin for prevention of morbidity and mortality in the six months following discharge from hospitals in western Kenya [9]. In this nested study, we sought to describe the proportion of phenotypic resistance to commonly used antibiotics, characterize β-lactamase genes among the phenotypically resistant isolates and assess the correlates of ESBL-producing *Klebsiella* isolates among children discharged from hospital in western Kenya.

## Methods

### Study design

In the parent trial [9], we systematically recruited children aged 1 – 59 months who were discharged from two county referral hospitals in Western Kenya between June 2016 and Nov 2019. In this nested cross-sectional study, we examine *Klebsiella* isolates collected at enrolment from two county hospitals in Western Kenya. The Kisii Teaching and Referral Hospital is located within the urban center in Kisii town whilst the Homa Bay County Teaching and Referral Hospital is in Homa Bay county. Kisii Teaching and Referral Hospital serves a population of about 1.2 million people with about 220,000 children under five years of age and serves as a major referral hospital in western Kenya [10]. Homa Bay County Teaching and Referral Hospital is classified as a level four healthcare institution, serving a predominantly rural population of around 1.1 million people. Homa Bay county has one of the highest under-five childhood mortality rates and HIV prevalence in the country [11].

Eligibility criteria included children who weighed at least 2 kg, had been hospitalized, recovered, and discharged from hospital, planned to remain in the study area for at least 6 months, had no contradiction to azithromycin, and had not been prescribed any macrolide antibiotics. We excluded children from the study if their hospital admission was solely due to trauma, injury, or birth defect, or if the legal guardian refused consent [9].

Prior to randomization, stool samples were collected from children, processed, and archived. Data on demographics, medical history, underlying medical conditions, clinical examination, and nutritional anthropometry were collected on standardized paper questionnaires by trained study clinicians. In the current nested cross-sectional study, we utilized a random sample of 245 children whose enrollment stool samples had *Klebsiella* isolated, linked them to clinical data recorded during hospital stay, and demographic and social economic factors collected from the primary caregiver during enrollment.

### Sample collection and processing

At enrollment, all children provided whole stool samples, or rectal swabs were used if whole stool collection was not feasible [9]. These samples were preserved in Cary-Blair media to ensure bacterial viability during transportation for microbiological culture. The samples were then promptly shipped to the central laboratory at the Kenya Medical Research Institute-Centre for Microbiology Research (KEMRI-CMR) in Nairobi within a 24-h timeframe. A swab or a sample of stool was streaked on MacConkey (MAC) (Oxoid, United Kingdom) and Eosin Methylene Blue agars (Oxoid, United Kingdom) and incubated in ambient air at 37 °C for 24 h. Morphologically distinct lactose fermenting mucoid colonies were subcultured onto Mueller Hinton (Oxoid, United Kingdom) agar and subjected to API 20E system (bioMérieux, Inc., France) and oxidase reactions for confirmation of *Klebsiella* spp. Confirmed *Klebsiella* spp. isolates were stocked in tryptone soy broth supplemented with 15% glycerol (Oxoid, United Kingdom) and frozen at -80 °C. For this analysis, the *Klebsiella* spp. isolates were thawed, quadrant streaked for isolation onto MAC agar and incubated at 37° C in ambient air to perform antimicrobial susceptibility testing (AST), DNA extraction and genetic characterization.

### Antibiotic susceptibility testing

The antibiotic susceptibility profiles of the *Klebsiella* isolates were determined by the Kirby-Bauer disk diffusion method as described by CLSI [12]. The antibiotics panels used included ceftriaxone (CRO, 30 µg), ceftazidime (CAZ, 30 µg), cefotaxime (CTX, 30 µg), cefoxitin (FOX, 30 µg), chloramphenicol (C, 30 µg), ciprofloxacin (CIP, 5 µg), gentamicin (CN,10 µg), amoxicillin-clavulanate (AMC, 20 µg/10 µg), meropenem (MEM, 10 µg), imipenem (IPM, 10 µg), azithromycin (AZM, 15 µg), and aztreonam (AZT, 30 µg). Zone diameters, measured in millimeters, established by CLSI-2020 M-100 were used to determine susceptibility, resistance, or an intermediate designation [12]. Both intermediate and resistant isolates were classified as non-susceptible [12].

### Determination of ESBL–producing *Klebsiella* spp

ESBL production was determined using the double-disc diffusion synergy test, which utilizes cefotaxime and ceftazidime with and without clavulanic acid [12]. The discs were placed 20 mm apart on a lawn culture of *Klebsiella* spp. plated on MH agar and incubated at 37 °C for 24 h as described previously [12, 13]. Quality control was assured by simultaneously plating and testing an ESBL-producing *Klebsiella* strain (ATCC 700603) and an ESBL-negative *E. coli* strain (ATCC 25922) [13]. ESBL-producing *Klebsiella* spp. was confirmed if the difference in the zone size between cefotaxime and the zone size of cefotaxime with clavulanic acid was ≥ 5 mm or if the difference in the zone size between ceftazidime and the zone size of ceftazidime with clavulanic acid was ≥ 5 mm as established previously [12].

### Genotypic detection of ESBL genes using conventional PCR

Bacterial DNA was extracted from ESBL-producing colonies of *Klebsiella* spp. using a boiling method. An inoculating loop was placed into bacteria pooled from an overnight culture in MH mixed with 0.5 ml nuclease free water. The cell suspension was heated for 10 min at 100 °C then centrifuged at 15,000 revolutions per minute for 5 min (maintained at 25 °C). The supernatant was used as DNA template for amplification. Extracted DNA was amplified using sets of primers targeting ESBL encoding genes (*bla*_*TEM*_*, bla*_*SHV*_*, bla*_*CTX-M*_*, bla*_*OXA*_) as previously described [14–16]. Briefly, a final reaction volume of 25 µl was used in a master mix containing 0.5 µl forward primer (0.2 µM), 0.5 µl reverse primer (0.2 µM), 9.5 µl nuclease free water. A 12.5 µl mix which included Taq DNA polymerase (2.5 units), 1 × PCR Buffer, MgCl_2_ (0.2 µM), and ultrapure dNTPs (200 µM,) followed by addition of 2 µl template DNA was combined with the PCR master mix. Amplification conditions consisted of 30 cycles of 94°C for 30 s, 50°C, 55 C and 60°C for 30 s, initial extension of 68°C for 1 min and with a final extension step of 68 °C for 5 min [16]. Gel electrophoresis of PCR products was carried out at 200 V on a 1.5% agarose gel, stained with gel red stain and visualized on a Vilber E-Box gel documentation system. All PCR reactions were run with both negative and positive DNA control templates.

### Definitions

Detailed descriptions of exposure variables and the derived variables has been provided elsewhere [9]. Briefly, we collected data on sex, child age, study site, HIV exposure, nutritional status, history of exclusive breast feeding, childhood vaccination (included pneumonia, rotavirus, measles, DPT, and BCG), length of hospital stay, antibiotic use during hospitalization, caregiver reported income, caregiver education level, household toilet type, water source and treatment, and household crowding. A household had access to improved water if the caregiver reported access to reliable piped water in the dwelling or community, or if the household primarily used water from a borehole, a protected spring, a well with a pump, bottled water, or rainwater from storage tanks for household chores. Access to treated drinking water was defined as a household whose drinking water is filtered, boiled, or chlorinated before use. Household crowding was defined as a household with more than two individuals sharing a room. The 2006 WHO growth references for children age < 5-years were used to construct anthropometric z-scores. We defined underweight as weight-for-age z-score (WAZ) less than -2SD, stunting as a height-for-age z-score (HAZ) less than -2SD, and wasting as weight-for-height/length z-score (WHZ) less than -2SD. Data on vaccination was derived from childhood vaccination cards if the cards were available at the hospital. However, if the cards were not available, the caregiver provided a report on the child’s vaccination status including the doses taken thus far. We derived an overall vaccine variable that defined children who had completed all essential age-appropriate vaccines, herein referred to “complete age-appropriate vaccination”.

### Statistical analysis

We reported the proportion of *Klebsiella* spp. isolates resistant to each tested antibiotic and carrying ESBL. To evaluate correlates of ESBL-producing *Klebsiella* spp., we constructed univariate and multivariable Poisson regression models with a robust variance for various child, hospital, and household factors, adjusting for key a priori confounders (age, sex, and site). Associations were considered statistically significant at an alpha < 0.05. All statistical analyses were performed in Stata (Version 17.0, Stata Corp, College Station, TX, USA).

## Results

### Baseline characteristics

**Fig. 1:**
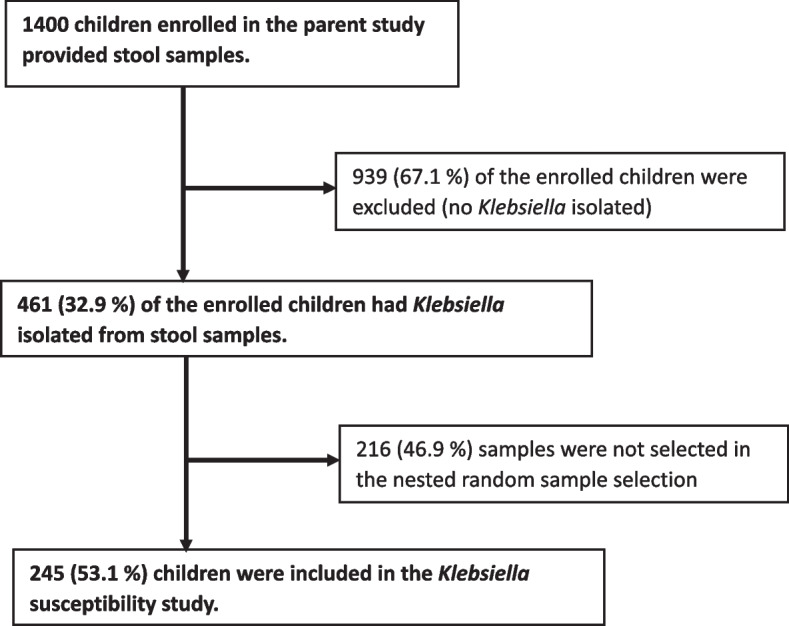
Participant flow chart

Out of 1400 children enrolled in the parent trial, 461/1400 (32.9%) had *Klebsiella* spp*.* isolated from their stool samples and 245 of those with *Klebsiella* spp. isolated, were randomly chosen to be included in this sub-study (Fig. 1). The 245 children had a median age of 15 months (IQR 8–30), 144/245 (58%) were from the Kisii site, 140/245 (57%) were male, 20/245 (8%) had severe wasting, and 18/245(7%) had moderate wasting. Diagnosis at discharge included 57/245 (23%) pneumonia, 19/245 (8%) diarrhea and 41/245 (17%) malaria cases. During hospitalization, 219/245 (89%) children had taken at least one antibiotic and 172/245 (70%) had taken more than 1 antibiotic: 77/245 (31%) received ceftriaxone, 2/245 (1%) received ciprofloxacin, 5/245 (2%) received cefuroxime, 142/245 (58%) received gentamicin, 19/245 (8%) received chloramphenicol, and 165/245 (67%) received penicillin. Further detailed description of the participants characteristics is shown by site in (Table S1).

### Phenotypic and genetic AMR

**Fig. 2:**
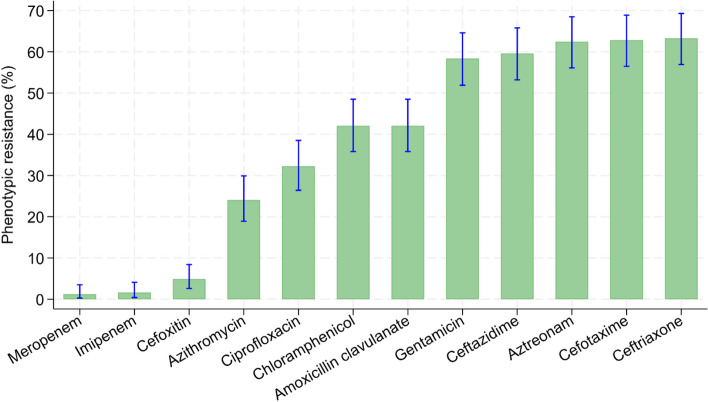
Prevalence of phenotypic resistance in *Klebsiella* isolates from children discharged from hospital in western Kenya. The error bars represent 95% CI

Overall, 231/245 (94.3%) were *K. pneumoniae* and 14/245 (5.7%) were *K. oxytoca*. A total of 154/245 (62.8%) isolates were phenotypically ESBL positive, 148/231 (64.1%) *K. pneumoniae* and 6/14 (42.9%) *K. oxytoca.* More than half, 152/245 (62%) harbored *Klebsiella* spp. isolates that were resistant to a third-generation cephalosporin; more specifically, 155/245 (63.2%) were resistant to ceftriaxone, 154/245 (62.8%) were resistant to cefotaxime and 146/245 (59.6%) were resistant to ceftazidime. A total of 152/245 (62%) *Klebsiella* isolates were non susceptible to at least three categories of antimicrobials considered as multidrug resistant (MDR). Resistance to cefoxitin was only 12/154 (4.9%) and 143/245 (58.0%) were resistant to gentamicin. Furthermore, among the less commonly prescribed antibiotics in Kenyan hospitals, 103/245 (42%) were resistance to chloramphenicol, 79/245 (32%) to ciprofloxacin and 59/245 (24%) to azithromycin. In contrast, only 4 isolates were resistant carbapenem antibiotics (meropenem or imipenem) (Fig. 2).

**Fig. 3:**
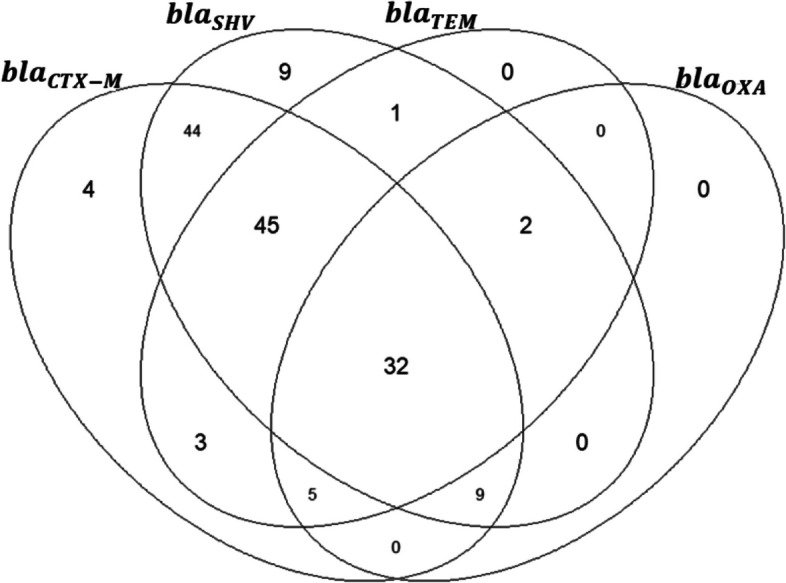
Venn diagram showing co-carriage of resistance genes among ESBL positive *Klebsiella* isolates

At least one ESBL-conferring gene was detected in all the 154 ESBL-producing *Klebsiella* isolates genotyped. The *bla*_CTX-M_ 142/154 (92.2%) and *bla*_SHV_ 142/154 (92.2%) were the most prevalent ESBL-conferring gene followed by *bla*_TEM_ 88/154 (57.1%) and *bla*_OXA_ 48/154 (31.2%). Over 90% (141/154) of the ESBL positive samples had multiple genetic markers, further details on co-carriage of genetic markers of resistance are shown in Table 1 segregated by species (Table 1). A total of 32/154 (20.8%) isolates co-carried all the 4 *bla* genes screened while majority of the isolates 61/154 (39.6%) had co-carriage of 3 β-lactamase genes. Only 13 isolates had either *bla*_CTX-M_ or *bla*_SHV_ as the only β-lactamase genes present (Fig. 3). A further description of the phenotypic resistance against gene carriage among isolates that were positive for ESBL is shown in Table 2.

**Table 1:** Co-carriage of resistance genes among ESBL positive Klebsiella isolates segregated by *Klebsiella *species

Only one ESBL encoding gene	*K. pneumoniae* (*N* = 148)	*K. oxytoca* (*n* = 6)	Total
*bla*_CTX-M_	4	0	4/154
*bla*_SHV_	9	0	9/154
*bla*_TEM_	0	0	
*bla*OXA	0	0	
Combination of 4 encoding gene			
*bla*_CTX-M + SHV + TEM + OXA_	30	2	32/154
Combination of 3 encoding gene			
*bla*_CTX-M + SHV + TEM_	45	0	45/154
*bla*_SHV + TEM + OXA_	2	0	2/154
*bla*_CTX-M + TEM + OXA_	4	1	5/154
*bla*_CTX-M + SHV + OXA_	7	2	9/154
Combination of 2 ESBL encoding Genes			
*bla*_CTX-M + SHV_	43	1	44/154
*bla*_CTX-M + TEM_	3	0	3/154
*bla*_SHV + TEM_	1	0	1/154

**Table 2:** Descriptive data on antimicrobial susceptibility testing and Beta lactamase genes among isolates that tested positive for ESBL

	**ESBL Genes**							
	***bla***_**CTX-M**_		***bla***_**SHV**_		***bla***_**TEM**_		***bla***_**OXA**_	
	Negative	Positive	Negative	Positive	Negative	Positive	Negative	Positive
	*N* = 12	*N* = 142	*N* = 12	*N* = 142	*N* = 66	*N* = 88	*N* = 106	*N* = 48
**Chloramphenicol**								
Susceptible	5 (42%)	63 (44%)	6 (50%)	62 (44%)	19 (29%)	49 (56%)	41 (39%)	27 (56%)
Non susceptible	7 (58%)	79 (56%)	6 (50%)	80 (56%)	47 (71%)	39 (44%)	65 (61%)	21 (44%)
**Ciprofloxacin**								
Susceptible	4 (33%)	71 (50%)	6 (50%)	69 (49%)	36 (55%)	39 (44%)	61 (58%)	14 (29%)
Non susceptible	8 (67%)	71 (50%)	6 (50%)	73 (51%)	30 (45%)	49 (56%)	45 (42%)	34 (71%)
**Gentamicin**								
Susceptible	2 (17%)	17 (12%)	1 (8%)	18 (13%)	4 (6%)	15 (17%)	14 (13%)	5 (10%)
Non susceptible	10 (83%)	125 (88%)	11 (92%)	124 (87%)	62 (94%)	73 (83%)	92 (87%)	43 (90%)
**Ceftriaxone**								
Susceptible	1 (8%)	0 (0%)	0 (0%)	1 (1%)	1 (2%)	0 (0%)	1 (1%)	0 (0%)
Non susceptible	11 (92%)	142 (100%)	12 (100%)	141 (99%)	65 (98%)	88 (100%)	105 (99%)	48 (100%)
**Cefoxitin**								
Susceptible	11 (92%)	133 (94%)	10 (83%)	134 (94%)	62 (94%)	82 (93%)	99 (93%)	45 (94%)
Non susceptible	1 (8%)	9 (6%)	2 (17%)	8 (6%)	4 (6%)	6 (7%)	7 (7%)	3 (6%)
**Amoxicillin/ clavulanate**								
Susceptible	6 (50%)	54 (38%)	6 (50%)	54 (38%)	29 (44%)	31 (35%)	42 (40%)	18 (38%)
Non susceptible	6 (50%)	88 (62%)	6 (50%)	88 (62%)	37 (56%)	57 (65%)	64 (60%)	30 (62%)
**Aztreonam**								
Susceptible	1 (8%)	3 (2%)	0 (0%)	4 (3%)	1 (2%)	3 (3%)	2 (2%)	2 (4%)
Non susceptible	11 (92%)	139 (98%)	12 (100%)	138 (97%)	65 (98%)	85 (97%)	104 (98%)	46 (96%)
**Ceftazidime**								
Susceptible	2 (17%)	6 (4%)	1 (8%)	7 (5%)	4 (6%)	4 (5%)	7 (7%)	1 (2%)
Non susceptible	10 (83%)	136 (96%)	11 (92%)	135 (95%)	62 (94%)	84 (95%)	99 (93%)	47 (98%)
**Cefotaxime**								
Susceptible	1 (8%)	1 (1%)	0 (0%)	2 (1%)	2 (3%)	0 (0%)	1 (1%)	1 (2%)
Non susceptible	11 (92%)	141 (99%)	12 (100%)	140 (99%)	64 (97%)	88 (100%)	105 (99%)	47 (98%)
**Azithromycin**								
Susceptible	7 (58%)	103 (73%)	8 (67%)	102 (72%)	53 (80%)	57 (65%)	83 (78%)	27 (56%)
Non susceptible	5 (42%)	39 (27%)	4 (33%)	40 (28%)	13 (20%)	31 (35%)	23 (22%)	21 (44%)
**Imipenem**								
Susceptible	11 (92%)	139 (98%)	12 (100%)	138 (97%)	64 (97%)	86 (98%)	103 (97%)	47 (98%)
Non susceptible	1 (8%)	3 (2%)	0 (0%)	4 (3%)	2 (3%)	2 (2%)	3 (3%)	1 (2%)
**Meropenem**								
Susceptible	11 (92%)	141 (99%)	12 (100%)	140 (99%)	66 (100%)	86 (98%)	105 (99%)	47 (98%)
Non susceptible	1 (8%)	1 (1%)	0 (0%)	2 (1%)	0 (0%)	2 (2%)	1 (1%)	1 (2%)

### Risk factors of ESBL carriage among *Klebsiella* spp. isolates

In the multivariable regression model adjusted for a priori confounders (age, site, and sex), any antibiotic administration during hospitalization was associated with a significant increase in the likelihood of *ESB*L-producing *Klebsiella* spp. (aPR = 4.51; 95%CI: 1.79–11.37, *p* < 0.001). Among children who received at least one antibiotic during hospitalization, ESBL-producing *Klebsiella* spp. isolates were more likely to be found in children who received ceftriaxone (aPR = 1.42; 95%CI: 1.19–1.71, *p* < 0.001) and chloramphenicol (aPR = 1.28; 95%CI: 1.01–1.62, *p* = 0.042) but not gentamicin (aPR = 0.72; 95%CI: 0.60–0.87, *p* < 0.001) or penicillin (aPR = 0.77; 95%CI: 0.62–0.95, *p* = 0.014) during hospital admission (Table S2). Compared to children who stayed in the hospital for 3 days or less, a longer hospital stay (> 3 days) was associated with an increased risk of ESBL-producing *Klebsiella* spp. (aPR = 1.42; 95% CI: 1.14–1.77, *p* = 0.002)*.* Surprisingly, children residing in households with access to treated water were significantly associated with a higher likelihood of ESBL carriage (aPR = 1.38; 95%CI: 1.12–1.71, *p* < 0.003). Living in a crowded household, open defecation, nutritional status, HIV status and household reported income were not significantly associated with β-lactamase-producing *Klebsiella* spp. (Table S2).

## Discussion

Among children discharged from hospital in western Kenya with *Klebsiella* spp. isolated in stool samples, resistance to commonly used antibiotics was common, and over 60% were ESBL-producing. ESBL-conferring genes of high epidemiologic significance were found in ESBL-producing *Klebsiella* spp. isolates, most commonly *bla*_CTX-M_ and *bla*_SHV_. These findings highlight the burden of commensal AMR bacteria with the ability to cause infections in children discharged from hospitals in western Kenya and suggest the potential for disease and/or transmission during the period following discharge from hospital.

Antibiotics are an essential, and often life-saving tool for hospitalized children in settings of high infectious-disease related morbidity and mortality. Phenotypic AMR was associated with longer period of hospitalization and antibiotic use during hospitalization consistent with our previous findings [17, 18] and findings from studies conducted elsewhere [19]. We observed a high prevalence of non-susceptibility to 3^rd^ generation cephalosporins (cefotaxime, ceftriaxone and ceftazidime) in our study sites, as has been described elsewhere in SSA [20–22]. These antibiotics are widely used during hospitalization in the management of bacterial infections [23]. Empiric antibiotic treatment is critical to pediatric hospital care, particularly where access to diagnostic tools such as bacterial culture and antimicrobial susceptibility testing are limited or unavailable. Therefore, while antibiotic use is often unavoidable during a hospital stay, it could be that limiting time in hospital could reduce the likelihood of carriage of ESBL-producing *Klebsiella* spp. that could pose problems during the discharge period.

Children leaving the hospital may be carrying ESBL producing *Klebsiella* acquired during their hospital stay. The high rates of AMR acquisition in *Klebsiella* spp. during hospitalization are well established. For instance, 55% of the neonates admitted without ESBL in Kilifi, Kenya, acquired ESBL during hospitalization whilst only 10% had ESBL at admission to inpatient care [24]. Children may acquire ESBL-producing pathogens from nosocomial infections during hospital admission or through colonization of ESBL-producing bacteria resulting from antimicrobial selection pressure [18, 25, 26]. There was no significant association between ESBL-producing *Klebsiella* spp. and social-demographic factors including age, sex and site consistent with findings from other studies [27, 28]

The WHO recommends tailoring therapy to local AMR patterns in SSA, however, this is usually hindered by the lack of data about local antimicrobial susceptibility profiles due to a lack of reliable and consistent testing due to insufficient laboratory capacities [22]. As a result, hospitals in SSA rely on clinical syndromes and the administration of broad-spectrum antibiotics in the treatment of serious bacterial infections [22]. Evidence shows that while syndromic diagnosis has a high sensitivity of detection, it is associated with low specificity therefore driving higher than necessary consumption of antibiotics, a key factor in the selection of antimicrobial-resistant bacteria [29]. In this African setting, most children received antibiotic during hospitalization which may explain the high rates of ESBL carriage. This is a cause of concern considering that beta-lactams are the mainstay in the management of bacterial illnesses in SSA. Cephalosporins mediate co-selection pressure conferring resistance to facilitating horizontal transfer of resistance determinants non-beta lactam antibiotics that include aminoglycosides and quinolones [18, 30].

Phenotypic AMR was associated with antibiotic use and duration of hospitalization, suggesting either selection for antibiotic-resistant bacteria or exposure to ESBL-producing bacteria during hospitalization. The association between ceftriaxone use and ESBL carriage has been previously described in the same settings [18]. Use of ceftriaxone and chloramphenicol during the hospital stay was associated with a higher prevalence of ESBL while the use of gentamicin and penicillin was significantly associated with a lower prevalence of ESBL, likely due to ceftriaxone exposure in the comparison group. Resistance to chloramphenicol may have occurred through modification of antibiotic target sites or efflux pumps extrusion or carriage of plasmid mediated determinants which reduce the effectiveness of the antibiotic. Chloramphenicol is used as first-line treatment for typhoid in Kenya and other East African countries [19, 31]. Carbapenems are broad spectrum members of the β-lactam antimicrobials with an additional β-ring which makes the antibiotic potent against beta-lactamases, attaching to the penicillin binding proteins of the cell wall thereby resulting in bacterial cell death [32] and are therefore effective in the treatment of ESBL producing *Klebsiella*. Additionally, carbapenems are expensive and not commonly prescribed, especially in public hospitals hence they are less likely to develop resistance.

The percentage of decreased susceptibility to gentamicin (58.0%), a common first-line antibiotics recommended by the WHO in the treatment and management of severe bacterial infections in resource limited settings [21], was consistent with previous studies conducted in SSA [29]. We observed a high degree of susceptibility to cefoxitin likely because cephamycin antibiotics are less likely to be hydrolyzed by ESBLs [18, 33]. *Bla*_CTX-M_ and *bla*_SHV_-type ESBL were the most predominant ESBL gene variants detected in this area consistent with the global increase in *bla*_CTX-M_ type ESBLs witnessed in the last two decades [33–35]. The co-carriage of bla genes observed in this study is consistent with findings from other studies [34, 36, 37]. Intrestingly, we obaserved isolates that contained upto upto 4 β-lactamase genes inferring circulation of multiple plasmid [36] within the same genetic environment medaiting multidrug resistance phenotype. This phenomena has previouly been reported in other studies within the SSA and other parts of the world [34, 36–38].

Antibiotic resistant bacteria may enter water sources from wastewater released from hospitals, household, or agricultural farmland [39]. The finding that children who have access to treated drinking water are at a higher risk of β-lactamase-producing *Klebsiella* spp. is surprising. We hypothesize that households that reported access to treated drinking water were probably situated in areas linked to water supply systems that are vulnerable to contamination [40], particularly in urban or peri-urban settings. Alternatively, whilst most countries in SSA recommend the use of chlorine in the treatment of water [41], studies have reported that chlorinated drinking water may contribute to the enrichment of antibiotic resistance bacteria and therefore aid the spread of antibiotic resistance genes [42–44].

An important strength of our study is that few studies provide a comprehensive assessment of AMR among children discharged from hospital who are at a high risk of morbidity and mortality. The study was conducted in a rural and peri-urban setting in western Kenya and therefore provides important data on AMR from a low-income country with a high burden of bacterial infection. Our study also had important limitations. In recruiting children discharged from hospital, we excluded those that died during hospitalization, a population potentially at the highest risk of AMR carriage. Therefore, the burden of ESBL in *Klebsiella* spp. isolates that was observed is likely an underestimate of the true burden among hospitalized children. This analysis included only children with *Klebsiella* isolated thus selecting for patients with high amounts of healthcare exposure introducing a potential limitation on generalizability. We only collected stool samples at the point of hospital discharge, making it difficult to distinguish between AMR acquired during hospitalization and that acquired before admission. Furthermore, we are not able to definitively determine how resistance to the antibiotic classes was acquired. To do this, targeted PCR analysis or whole genome sequencing would be required. Our study was conducted in two counties of western Kenya and therefore difficult to generalize to other settings. Finally, we cannot exclude the possibility that the identified risk factors may be explained by confounders not accounted for in the analysis. Potential confounders may include unmeasured sociodemographic factors.

## Conclusion

Carriage of β-lactamase producing *Klebsiella* spp. is common among children discharged from hospital in western Kenya and is associated with the duration of hospitalization and antibiotic use. The findings emphasize the need for continued monitoring of antimicrobial susceptibility profiles to inform the development and implementation of appropriate treatment guidelines. Measures beyond antimicrobial stewardship that include infection control within hospitals are needed to mitigate the spread and impact of β-lactamase-producing *Klebsiella* on the public health system. Efforts to improve diagnosis and detection of AMR pathogens within the healthcare system is needed to inform personalized therapeutics in these settings. Furthermore, research is needed to investigate the quality of treated drinking water and examine the effectiveness of the technologies used in the treatment of drinking water in these settings to inform public health policy change.

### Supplementary Information

**Supplementary Material 1. **

**Supplementary Material 2. **

## Data Availability

All datafiles are available from Harvard Dataverse (https://doi.org/10.7910/DVN/CEJVKZ).

## Abbreviations

AMR: Antimicrobial Resistance
AST: Antimicrobial Susceptibility Testing
ATCC: American Type Culture Collection
*Bla*: Beta Lactamase
CLSI: Clinical Laboratory Standard Institute
ESBL: Extended Spectrum Beta Lactamase
PCR: Polymerase Chain Reaction
WHO: World Health Organization
SSA: Sub Saharan Africa
LMIC: Low- and Middle-Income Countries
KEMRI: Kenya Medical Research Institute
