# Supplementary material for: Phenotypic and molecular characterization of β-lactamase-producing Klebsiella species among children discharged from hospital in Western Kenya
Source: BMC Microbiol. 2024 Apr 23;24:135. doi: 10.1186/s12866-024-03284-7 (PMC11040804; doi:10.1186/s12866-024-03284-7)
Supplement: Supplementary file 1 — Supplementary Material 1. [file 12866_2024_3284_MOESM1_ESM.pdf]

Table S1: Characteristics of children enrolled in the study.

	KISII N=144 (iii)	HOMABAY N=101 (iii)	Total N=245 (iii)
**Child Characteristics**			
Age (months)			
0-5	31 (22%)	9 (9%)	40 (16%)
6-11	31 (22%)	26 (26%)	57 (23%)
12-23	33 (23%)	32 (32%)	65 (27%)
24-59	49 (34%)	34 (34%)	83 (34%)
Sex			
Male	86 (60%)	54 (53%)	140 (57%)
Female	58 (40%)	47 (47%)	105 (43%)
Breastfeeding[i]			
Exclusively breastfed	43 (30%)	66 (65%)	109 (44%)
Partially breastfed	86 (59%)	35 (35%)	121 (50%)
Unknown	15 (10%)	0 (0%)	15 (6%)
Child HIV status [ii]			
HIV unexposed	134 (93%)	70 (69%)	204 (83%)
HIV exposed, uninfected/infection status unknown	8 (6%)	30 (30%)	38 (16%)
Underweight (WAZ < -2)			
WAZ ≥ -2	117 (81%)	87 (86%)	204 (83%)
WAZ <-2	27 (19%)	14 (14%)	41 (17%)
Stunting (HAZ/LAZ < -2)			
HAZ ≥ -2	99 (69%)	82 (82%)	181 (74%)
HAZ <-2	44 (31%)	18 (18%)	62 (26%)
Acute malutrition			
None	123 (85%)	84 (83%)	207 (84%)
MAM	8 (6%)	10 (10%)	18 (7%)
SAM	13 (9%)	7 (7%)	20 (8%)
**Vaccination Status [iv]**			
Complete essential vaccination for age			
No	79 (55%)	69 (68%)	148 (60%)
Yes	65 (45%)	32 (32%)	97 (40%)
**Hospitalization Information**			
Length of hospital stay			
<4 days	54 (38%)	48 (48%)	102 (42%)
≥4 days	87 (62%)	53 (52%)	140 (58%)
Discharge diagnosis			
Diarrhoea	10 (7%)	9 (9%)	19 (8%)
URTI	3 (2%)	1 (1%)	4 (2%)
Pneumonia	39 (27%)	18 (18%)	57 (23%)
Malaria	18 (13%)	23 (23%)	41 (17%)
Malnutrition	12 (8%)	7 (7%)	19 (8%)

Other diseases	60 (42%)	43 (43%)	103 (42%)
Any antibiotic used during admission			
No	6 (4%)	20 (20%)	26 (11%)
Yes	138 (96%)	81 (80%)	219 (89%)
Ceftriaxone used during admission [v]			
No	106 (74%)	62 (61%)	168 (69%)
Yes	38 (26%)	39 (39%)	77 (31%)
Ciprofloxacin used during admission [v]			
No	144 (100%)	99 (98%)	243 (99%)
Yes	0 (0%)	2 (2%)	2 (1%)
Cefuroxime used during admission [v]			
No	140 (97%)	100 (99%)	240 (98%)
Yes	4 (3%)	1 (1%)	5 (2%)
Gentamicin used during admission [v]			
No	42 (29%)	61 (60%)	103 (42%)
Yes	102 (71%)	40 (40%)	142 (58%)
Chloramphenicol used during admission [v]			
No	127 (88%)	99 (98%)	226 (92%)
Yes	17 (12%)	2 (2%)	19 (8%)
Penicillin used during admission [v]			
No	23 (16%)	57 (56%)	80 (33%)
Yes	121 (84%)	44 (44%)	165 (67%)
**Household Information**			
Caregiver reported income			
Income ≥ 5000 ksh	50 (35%)	17 (17%)	67 (27%)
Income < 5000 ksh	90 (63%)	74 (73%)	164 (67%)
Unknown or refuse to answer	4 (3%)	10 (10%)	14 (6%)
Crowding (>2 persons per room)			
No	98 (68%)	32 (32%)	130 (53%)
Yes	46 (32%)	69 (68%)	115 (47%)
Improved water source			
No	25 (17%)	28 (28%)	53 (22%)
Yes	119 (83%)	73 (72%)	192 (78%)
Treated drinking water			
No	78 (56%)	23 (23%)	101 (42%)
Yes	62 (44%)	76 (77%)	138 (58%)
Toilet			
Private for household only	81 (57%)	35 (35%)	116 (48%)
Shared with ≥1 other household	61 (43%)	53 (52%)	114 (47%)
Open defecation	1 (1%)	13 (13%)	14 (6%)

Abbreviations: HIV - Human Immunodeficiency Virus; HAZ- height-for-age; LAZ- length-for-age MAM- moderate acute malnutrition; SAM-severe acute malnutrition; WAZ- weight-for-age; URTI – upper respiratory tract infection

Footnotes

i. Whether the child is currently breastfeeding (≤6 months old) or if the caregiver practiced breastfeeding when the child was under 6 months old.

ii. Six children had exposure and infection status unknown and were excluded.

iii. Column percentages

iv. Completed all essential vaccination defined as having complete vaccination for Pneumococcal, Rotavirus, DPT, Measles and BCG up until the age of the child allowing a 2-week margin.

v. Include both children who received any antibiotic and those that did not receive any antibiotic during hospitalization.
