# Supplementary material for: Phenotypic and molecular characterization of β-lactamase-producing Klebsiella species among children discharged from hospital in Western Kenya
Source: BMC Microbiol. 2024 Apr 23;24:135. doi: 10.1186/s12866-024-03284-7 (PMC11040804; doi:10.1186/s12866-024-03284-7)
Supplement: Supplementary file 2 — Supplementary Material 2. [file 12866_2024_3284_MOESM2_ESM.pdf]

Appendix II

Table S2: Predictors of ESBL in *Klebsiella* isolated from stool samples of the participating children. Model 1 and Model 2 presents results from the univariable Poisson regression and the adjusted multivariable Poisson regression model (adjusted for age, sex, and site only) respectively with robust standard errors. The "**ESBL +"** and "**ESBL -"** denote the presence and absence of the **ESBL** in the isolated *Klebsiella* from stool samples.

	**ESBL+** N=154 (%) [iii]	**ESBL-** N=91 (%) [iii]	Model 1			Model 2		
			**PR** [iv]	**95% CI**	**p-value**	**Adj. PR** [iv]	**95% CI**	**p-value**
Location of the facility								
KISII	96 (62%)	48 (53%)	Ref					
HOMABAY	58 (38%)	43 (47%)	0.86	(0.70, 1.06)	0.152	0.85	(0.68, 1.03)	0.092
**Child Characteristics**								
Age (months)	14 (9-29)	18 (7-36)	1.00	(0.99, 1.01)	0.696	1.00`	(0.99, 1.01)	0.722
Age (months)								
0-5	23 (15%)	17 (19%)	Ref					
6-11	37 (24%)	20 (22%)	1.13	(0.81, 1.57)	0.469	1.18	(0.84, 1.64)	0.342
12-23	45 (29%)	20 (22%)	1.20	(0.88, 1.65)	0.244	1.26	(0.92, 1.73)	0.152
24-59	49 (32%)	34 (37%)	1.03	(0.74, 1.42)	0.872	1.06	(0.77, 1.46)	0.734
Sex								
Male	88 (57%)	52 (57%)	Ref					
Female	66 (43%)	39 (43%)	1.00	(0.82, 1.22)	1.000	1.02	(0.84, 1.46)	0.874
Breastfeeding [i]								
Exclusively breastfed	60 (39%)	49 (54%)	Ref					
Partially breastfed	84 (55%)	37 (41%)	1.26	(1.03, 1.55)	0.028	1.20	(0.96, 1.50)	0.107
Unknown	10 (6%)	5 (5%)	1.21	(0.81, 1.80)	0.344	1.15	(0.76, 1.73)	0.502
Child HIV status [ii]								

HIV unexposed	129 (85%)	75 (83%)	Ref					
HIV positive or exposed	23 (15%)	15 (17%)	0.96	(0.72, 1.26)	0.757	1.08	(0.81, 1.46)	0.590
Underweight (WAZ < -2)								
WAZ ≥ -2	130 (84%)	74 (81%)	Ref					
WAZ <-2	24 (16%)	17 (19%)	0.92	(0.70, 1.21)	0.550	0.89	(0.67, 1.17)	0.399
Stunting (HAZ/LAZ < -2)								
HAZ ≥ -2	114 (75%)	67 (74%)	Ref					
HAZ <-2	38 (25%)	24 (26%)	0.97	(0.78, 1.22)	0.815	0.96	(0.76, 1.21)	0.728
Acute malnutrition								
None	128 (83%)	79 (87%)	Ref					
MAM	12 (8%)	6 (7%)	1.08	(0.76, 1.52)	0.669	1.07	(0.75, 1.52)	0.700
SAM	14 (9%)	6 (7%)	1.13	(0.83, 1.54)	0.428	1.07	(0.78, 1.47)	0.670
**Vaccination Status** [v]								
Complete essential vaccination for current age								
No	90 (58%)	58 (64%)	Ref					
Yes	64 (42%)	33 (36%)	1.08	(0.89, 1.32)	0.408	1.07	(0.88, 1.29)	0.504
**Hospitalization Information**								
Length of hospital stay								
<4 days	51 (34%)	51 (57%)	Ref					
≥4 days	101 (66%)	39 (43%)	1.44	(1.16, 1.80)	0.001	1.42	(1.14, 1.77)	0.002
Any antibiotic used during admission [vi]								
No	4 (3%)	22 (24%)	Ref					
Yes	150 (97%)	69 (76%)	4.45	(1.80,11.04)	**0.001**	4.51	(1.79,11.37)	**0.001**
Ceftriaxone use during admission [vi]								
No	85 (57%)	57 (83%)	Ref					
Yes	65 (43%)	12 (17%)	1.43	(1.21, 1.69)	**<0.001**	1.42	(1.19, 1.71)	**<0.001**
Gentamicin use during admission [vi]								
No	64 (43%)	13 (19%)	Ref					

Yes	86 (57%)	56 (81%)	0.72	(0.61, 0.85)	<0.001	0.72	(0.60, 0.87)	**<0.001**
Chloramphenicol use during admission [vi]								
No	134 (89%)	66 (96%)	Ref					
Yes	16 (11%)	3 (4%)	1.27	(1.02, 1.58)	0.034	1.28	(1.01, 1.62)	**0.042**
Penicillin use during admission [vi]								
No	44 (29%)	10 (14%)	Ref					
Yes	106 (71%)	59 (86%)	0.78	(0.66, 0.93)	0.005	0.77	(0.62, 0.95)	0.014
**Household Information**								
Caregiver reported income								
Income≥ 5000 ksh	44 (29%)	23 (25%)	Ref					
Income < 5000 ksh	103 (67%)	61 (67%)	0.96	(0.78, 1.18)	0.677	1.00	(0.81, 1.24)	0.976
Unknown or refuse to answer	7 (5%)	7 (8%)	0.76	(0.44, 1.32)	0.334	0.83	(0.47, 1.47)	0.520
Crowding (>2 persons per room)								
No	86 (56%)	44 (48%)	Ref					
Yes	68 (44%)	47 (52%)	0.89	(0.73, 1.09)	0.261	0.93	(0.75, 1.14)	0.472
Improved water source								
No	32 (21%)	21 (23%)	Ref					
Yes	122 (79%)	70 (77%)	1.05	(0.82, 1.34)	0.681	1.03	(0.81, 1.31)	0.836
Treated drinking water								
No	56 (37%)	45 (52%)	Ref					
Yes	96 (63%)	42 (48%)	1.28	(1.04, 1.58)	0.022	1.38	(1.12, 1.71)	0.003
Toilet								
Private, for household only	74 (48%)	42 (46%)	Ref					
Shared with ≥1 other household	71 (46%)	43 (47%)	0.98	(0.80, 1.19)	0.813	0.98	(0.80, 1.20)	0.847
Open defecation	8 (5%)	6 (7%)	0.90	(0.56, 1.44)	0.650	1.00	(0.60, 1.65)	0.993

Abbreviations: HAZ- height-for-age; WAZ- weight-for-age; LAZ- length-for-age; MAM- moderate acute malnutrition; SAM-severe acute malnutrition; Adj. Prevalence Ratio- adjusted prevalence ratio

Footnotes

i. *Whether the child is currently breastfeeding (≤6 months old) or if the mother practiced breastfeeding when the child was under 6 months old.*
ii. *Six children had exposure and infection status unknown and were excluded*
iii. *Column percentages*
iv. *PR is the Prevalence Ratio whilst "*Adj. PR" is the adjusted prevalence ratio (adjusted for sex, age and site)
v. *Completed all essential vaccination defined as having complete age-appropriate vaccination for Pneumococcal, Rotavirus, DPT, Measles and BCG up until the age of the child allowing a 2-week margin.*
vi. *Among children who received at least one antibiotic during hospitalization (antibiotic of interest vs. other antibiotics).*
